# Neoadjuvant short-course radiotherapy or chemoradiation plus consolidative chemotherapy followed by radical operation for locally advanced rectal cancer

**DOI:** 10.3389/fonc.2023.1284569

**Published:** 2024-01-23

**Authors:** Shing Fung Lee, Pui Lam Yip, Barry Wo, Natalie Sean-Man Wong, Balamurugan A. Vellayappan, Harvey J. Mamon, Francis Ann Shing Lee

**Affiliations:** ^1^ Department of Clinical Oncology, Tuen Mun Hospital, New Territories West Cluster, Hospital Authority, Hong Kong, Hong Kong SAR, China; ^2^ Department of Radiation Oncology, National University Cancer Institute, National University Hospital, Singapore, Singapore; ^3^ Department of Radiation Oncology, Dana-Farber Cancer Institute/Brigham and Women’s Hospital, Boston, MA, United States

**Keywords:** neoadjuvant therapy, rectal cancer, radiotherapy, chemotherapy, treatment response, survival

## Abstract

**Introduction:**

Limited evidence compares short-course radiotherapy (SCRT) and long-course chemoradiotherapy (LCCRT), both of which are followed by consolidative chemotherapy before radical rectal surgery. We conducted a retrospective cohort study to assess treatment response, survival outcomes, and toxicity in patients with locally advanced rectal cancer.

**Materials and methods:**

Patients (cT3–4 and/or N+) treated with SCRT or LCCRT, consolidative chemotherapy, or total mesorectal excision between 2013 and 2021 were identified. the cause-specific cumulative incidence of disease-related treatment failure, locoregional recurrence, distant metastases, and overall survival were evaluated using flexible parametric competing risk analysis and Kaplan–Meier methods, adjusted for treatment regimens and clinicopathological factors. A pathological complete response (pCR), tumor downstaging, and toxicity have been reported.

**Results:**

Among the 144 patients, 115 (80%) underwent curative rectal surgery. The LCCRT and SCRT groups achieved pCR in 10 (18%) and seven (12%) patients, respectively (odds ratio, 1.68; 95% confidence interval [CI], 0.59–4.78). The adjusted cause-specific hazard ratio for disease-related treatment failure with LCCRT versus SCRT was 0.26 (95% CI, 0.08–0.87). Three-year cumulative probability of disease-related treatment failure was 10.0% and 25.6% for LCCRT and SCRT, respectively. No significant differences in T-downstaging, N-downstaging, significant pathologic downstaging (ypT0-2N0), locoregional failure, distant metastasis, or overall survival were found. Late rectal toxicity occurred in 10 (15%) LCCRT and two (3%) SCRT patients, respectively.

**Conclusion:**

LCCRT with consolidative chemotherapy demonstrated improved disease-related treatment failure compared with SCRT, despite higher late rectal toxicity. Further research is needed to assess the long-term oncologic outcomes and toxicity.

## Introduction

Surgical resection is the primary treatment for early stage rectal cancer, whereas long-course chemoradiotherapy (LCCRT) followed by total mesorectal excision (TME) and selective adjuvant chemotherapy are recommended for locally advanced cases, offering improved local control compared with postoperative radiotherapy (1, 2). Traditional LCCRT involves 45 Gy–50.4 Gy in 25–28 daily fractions, concurrent chemotherapy, and delayed surgery (6–12 weeks after chemoradiation), resulting in prolonged treatment duration.

An alternative is neoadjuvant short-course radiotherapy (SCRT) with 25 Gy in five daily fractions (3, 4). SCRT followed by immediate surgery yields lower pathological complete response (pCR) rates and tumor downstaging than LCCRT (5). However, delaying surgery after SCRT improves these outcomes, as shown in the Stockholm III trial (5). The interval between SCRT and surgery presents an opportunity for additional chemotherapy, potentially eliminating micrometastases and enhancing radiotherapy and chemotherapy synergism for greater primary tumor downstaging.

Recently, SCRT, followed by consolidative chemotherapy, has emerged as a viable alternative to LCCRT. The Polish II study demonstrated higher R0 resection rates and overall survival for patients receiving SCRT and FOLFOX4 than for those receiving LCCRT (6). A phase 2 study reported a 25% pCR rate and 71% T-downstaging effect with SCRT and four cycles of mFOLFOX6 (7). Various randomized trials have found similar approaches to yield superior or non-inferior oncological outcomes compared with conventional LCCRT (8–10).

Some trials have compared SCRT with neoadjuvant chemotherapy versus LCCRT alone (6, 8, 9, 11), whereas others have assessed LCCRT plus neoadjuvant chemotherapy (12–15). These treatment regimens are heterogeneous, with variations in neoadjuvant and adjuvant chemotherapy cycles, making it unclear whether the response in the SCRT arm was due to the sequencing of multiagent chemotherapy, radiotherapy dose, or fractionation. The effectiveness of SCRT compared with standard LCCRT in the context of total neoadjuvant therapy (TNT) or near TNT remains uncertain. Therefore, we conducted a retrospective study to compare the treatment response, survival outcomes, and toxicity between patients receiving SCRT and LCCRT, followed by consolidative chemotherapy before radical rectal surgery.

## Materials and methods

### Eligibility and assessments

We included patients aged >18 years with biopsy-proven cT3–4 primary rectal adenocarcinoma, with or without N1 or N2 status, and proximal extension ≤15 cm from the anal verge. Eligible participants were those who were medically fit for chemotherapy and considered candidates for radical rectal surgery upon adequate preoperative treatment response. Patients with metastatic disease, other malignancies, or intolerance to chemotherapy or surgery were excluded. The same eligibility criteria were applied to the LCCRT and SCRT groups.

The decision to allocate patients to SCRT or LCCRT was based on detailed magnetic resonance imaging (MRI) findings, which included tumor location, extent of mesorectal fascia involvement, and lymph node status. These treatment decisions were made during multidisciplinary team discussions to ensure a comprehensive and patient-specific approach to the treatment planning. Patients with a threatened mesorectal fascia, as determined by MRI, were generally inclined towards LCCRT, although this was ultimately an individualized decision.

Pre-treatment assessments included physical examinations: pelvic MRI, or computed tomography (CT) of the chest, abdomen, and pelvis (or 18F-fluorodeoxyglucose positron emission tomography-computed tomography [PET-CT]). A complete colonoscopy was performed when feasible. Otherwise, assessment of the colon proximal to the obstructive tumor was deferred if other imaging modalities showed no evidence of synchronic colonic tumors. T-stage evaluations were based on physical examination and MRI. The American Joint Committee on Cancer 7th and 8th editions have been used for cancer staging (16, 17). High-resolution T1-weighted, T2-weighted, T1-weighted with contrast, and diffused-weighted sequence MRI were mandatory before and after neoadjuvant treatment for radically resectable cases.

Post-treatment follow-up involved physical examination and carcinoembryonic antigen (CEA) measurement at each clinic visit. Patients were reviewed every three months for two years, then every four to six months from years three to five. Imaging was performed if recurrence was suspected or if CEA levels increased. Colonoscopy was performed within three years postoperatively and repeated within five years. In cases of incomplete preoperative colonoscopy, the first colonoscopy was performed within the first year after surgery.

### Treatment

SCRT involved 5 daily fractions over 5–7 days, using 3D conformal (3D-CRT) or intensity-modulated radiation therapy (IMRT) using 6-to 10-MV photons and symmetrical planning target volume (PTV) margins of 1 cm. Following the Radiation Therapy Oncology Group guidelines (11), the elective regional nodal target volume, the involved rectum, and the mesorectal compartment with 1 cm superior and inferior margins received 25 Gy. If the involved pelvic nodes extended beyond the primary tumor, the mesorectal coverage was adjusted accordingly. The gross tumor and clinically positive regional nodes in the pelvis could receive an integrated boost of up to 30 Gy using IMRT. A minimum PTV coverage of 95% by the prescription dose was required, with a maximum allowed dose of 108% of the prescription dose. The small bowel was limited to ≤150 cc and ≥45 Gy (equivalent dose in 2 Gy fractions [EQD2]) and the bowel bag to ≤200 cc and ≥45 Gy (EQD2).

In the LCCRT cohort, target volumes were defined as previously described, receiving 50.4 Gy in 28 fractions. Boost doses of 3.6 Gy–5.4 Gy (1.8 Gy per fraction) were delivered to the gross tumor and clinically positive regional nodes in the pelvis, either with integrated boost using IMRT or sequentially with IMRT or 3D-CRT. Concurrent single-agent chemotherapy was administered: capecitabine 825 mg/m^2^ orally twice daily during RT or 5-fluorouracil 500 mg/m^2^ intravenously on days 1–3 and 29–31.

Both the SCRT and LCCRT groups received consolidative CAPOX chemotherapy after completing radiotherapy with 2–3 weeks rest. Chemotherapy consisted of CAPOX (capecitabine 1,000 mg/m² orally twice daily on days 1–14, oxaliplatin 130 mg/m² intravenously on day 1, and a chemotherapy-free interval between days 15 and 21) or modified FOLFOX6 (oxaliplatin 85 mg/m² intravenously on day 1, leucovorin 400 mg/m² intravenously on day 1, followed by a bolus of 5-fluorouracil 400 mg/m² intravenously on day 1 and 5-fluorouracil 2,400 mg/m² intravenously for 46 h on days 1 and 2, followed by a chemotherapy-free interval between days 3 and 14). Standard dose modifications were performed.

Patients were scheduled for surgery 8–12 weeks post-radiation if MRI reassessment showed tumor response and the disease was deemed resectable. Radical rectal surgery involves TME as part of low anterior resection or abdominoperineal resection, with more extensive resection (including exenteration, left colectomy, hysterectomy, or cystectomy) performed in some cases. Adjuvant chemotherapy, although not mandated, was considered during multidisciplinary team meetings and could include oxaliplatin-based doublet regimens (CAPOX or modified FOLFOX6) or single-agent capecitabine or 5-fluorouracil.

### Toxicity assessments

Side effects were evaluated using the National Cancer Institute Common Terminology Criteria for Adverse Events, version 4.0. Preoperative toxicity spanned from the start of neoadjuvant treatment to the date of rectal surgery, while postoperative complications occurred from the date of surgery to 30 days post-surgery and were classified according to the Clavien–Dindo classification (18). Late toxicity was determined from 30 days post-surgery until the last follow-up, recurrent rectal cancer, or death, whichever occurred first.

### Study outcomes

Disease-related treatment failure was assessed and defined as the first occurrence of locoregional failure, distant metastasis, a new primary colorectal tumor, or rectal cancer/treatment-related death (9). It was calculated from the time of radiotherapy to the endpoint or censoring date, whichever occurred earlier. Locoregional failure and distant metastasis were analyzed as separate outcomes. Locoregional failure includes locally progressive disease leading to an unresectable tumor, local R2 resection, or locoregional recurrence after R0–R1 resection (9). Overall survival was also assessed (time from radiotherapy initiation to any cause of death). Other outcomes included pCR (no residual tumor on pathological assessment after definitive surgery), treatment toxicities, and surgical complications within 30 days (9, 19).

### Statistical analysis

Descriptive statistics were generated for the demographics, follow-up duration, and prevalence of characteristics in the SCRT and LCCRT groups. Continuous variables were presented as medians with interquartile ranges or means with standard deviations and compared using t-tests or rank-sum tests, depending on distributions. Categorical variables are presented as percentages and compared using Fisher’s exact test or χ^2^ test. The cumulative incidence of disease-related treatment failure was calculated, accounting for nontreatment-related deaths as a competing risk. Cumulative incidences of distant metastases and locoregional failure were calculated, accounting for all-cause death as a competing risk (20–23). For all competing risk analyses, adjusted cause-specific hazard ratios (HRs) were calculated using a flexible parametric survival model, accounting for the inverse probability of censoring and treatment weights (20, 21, 24–26). Overall survival was estimated using the Kaplan–Meier method (27, 28). pCR odds ratios (ORs) and 95% confidence intervals (CIs) were calculated. Patients alive without reaching the specified endpoints by 1 May 2022, were censored. Statistical analyses and survival model fitting were conducted using Stata v.16.1 (StataCorp, College Station, TX) (29, 30). A two-tailed *P* of <0.05 was considered statistically significant. This study was approved by the Research Ethics Committee, New Territories West Cluster, Hospital Authority, Hong Kong (reference number: NTWC/REC/19054). 

## Results

### Description of the cohort

Between 2013 and 2021, 144 patients underwent neoadjuvant radiotherapy for rectal cancer (**Table 1**). The median follow-up duration was 3.1 years (interquartile range [IQR], 1.8–4.5 years). The median age at diagnosis was 63 years (IQR, 57–69 years), with 83% males. Twenty-nine (20%) patients had cT4 tumors, 115 (80%) had cT3, and 129 (90%) tumors had cN+. Ninety-four (65%) and 23 (16%) patients had involved and threatened (≤1 mm) mesorectal fascia, respectively, on MRI. Thirty-five patients (24%) showed extramural venous invasion (EMVI).

**Table 1:** Patients and treatment characteristics, 2013–2021 (N = 144).

Characteristics	All patients (N = 144)	SCRT (n = 78)	LCCRT (n = 66)	P^*^
**Age, year, median (IQR)**	63 (57–69)	65 (59–75)	62 (55–67)	0.001
**Sex, n (%)**				0.496
Male	119 (83)	66 (85)	53 (80)	
Female	25 (17)	12 (15)	13 (20)	
**Performance status (ECOG), n (%)**				0.003
0/1	121 (84)	59 (76)	62 (94)	
2	23 (16)	19 (24)	4 (6)	
**RCS co-morbidity scores, n (%)**				0.172
0	117 (81)	59 (76)	58 (88)	
1	20 (14)	14 (18)	6 (9)	
≥2	7 (5)	5 (6)	2 (3)	
**Combined clinical stage, n (%)**				0.538
II	15 (10)	7 (9)	8 (12)	
III	129 (90)	71 (91)	58 (88)	
**Clinical T stage, n (%)**				0.005
cT3	115 (80)	69 (88)	46 (70)	
cT4	29 (20)	9 (12)	20 (30)	
**Clinical N stage, n (%)**				0.538
cN0	15 (10)	7 (9)	8 (12)	
cN+	129 (90)	71 (91)	58 (88)	
**Distance from anal verge (cm)**				
Median (IQR), cm	6.8 (4.7–8.5)	6.0 (4.8–8.0)	7.0 (4.5–9.1)	0.132
0–5 (lower)	51 (35.4)	32 (41.0)	19 (28.8)	0.058
>5–10 (mid)	76 (52.8)	41 (52.6)	37 (53.0)	
>10–15 (upper)	17 (11.8)	5 (6.4)	12 (18.2)	
**Mesorectal fascia, n (%)**				0.782
Involved	94 (65)	49 (63)	45 (68)	
Threatened (≤1 mm)	23 (16)	13 (17)	10 (15)	
>1mm	27 (19)	16 (21)	11 (17)	
**EMVI, n (%)**	35 (24)	26 (33)	9 (14)	0.006
**Dose to primary tumor, Gy, median (range)**	30.00 (27.50–50.40)	28.75 (25.00–30.00)	54.04 (50.40–56.00)	–
**Fraction size, Gy, range**	5.00 (1.93–6.00)	5.75 (5.00–6.00)	1.93 (1.80–2.00)	–
**Radiotherapy technique, n (%)**				<0.001
3D conformal	91 (63)	29 (37)	62 (94)	
IMRT	53 (37)	49 (63)	4 (6)	
**Surgery with curative intent after preoperative treatment, n (%)**	115 (80)	60 (77)	55 (83)	0.026
**Surgical technique, n (%)**				0.865
Sphincter preserving	87 (76)	45 (75)	42 (76)	
Sphincter non-preserving	28 (24)	15 (25)	13 (24)	
**Blood loss in the operation, ml, median (IQR)**	250 (150–500)	225 (100–400)	275 (150–500)	0.452
**Operation time, minutes, median (IQR)**	300 (260–358)	300 (256–353)	300 (271–373)	0.470
**Number of cycles of neoadjuvant chemotherapy, median (IQR)**	2 (2–4)	2 (2–4)	2 (2–5)	0.583
**Number of cycles adjuvant chemotherapy, median (IQR)**	4 (0–5)	4 (1–6)	4 (0–4)	0.007
**Time from commencement of radiotherapy to surgery, weeks, median (IQR)**	16 (14–20)	14 (11–17)	18 (16–29)	<0.001
**Time from completion of radiotherapy to surgery, weeks, median (IQR)**	12 (10–18)	13 (10–16)	12 (10–23)	0.787
**Pretreatment CEA, ug/L, median (range)**	8.6 (3.7–29.4)	8.0 (4.3–29.4)	9.0 (3.1–30.5)	0.360
**Preoperative CEA, ug/L, median (range)**	3.7 (2.5–6.6)	3.7 (2.7–6.6)	3.5 (2.4–6.6)	0.591
**Postoperative CEA, ug/L, median (range)**	2.3 (1.8–3.6)	2.4 (1.8–3.6)	2.1 (1.8–3.8)	0.305

3D, 3-dimensional; CEA, carcinoembryonic antigen; ECOG, Eastern Cooperative Oncology Group; EMVI, extramural venous invasion; Gy, Gray; IMRT, intensity modulated radiotherapy; IQR, interquartile range; LCCRT, long-course chemoradiotherapy; RCS, Royal College of Surgeons; SCRT, short-course radiotherapy.

^*^Tested by Chi-squared test or Wilcoxon test for association.

### Treatment delivery

Four patients in the LCCRT cohort experienced treatment interruptions. Patients received a median of two neoadjuvant chemotherapy cycles (IQR 2–4 cycles); 45 (58%) and 30 (39%) received CAPOX and capecitabine, respectively. Forty-one patients received adjuvant chemotherapy, and 26 (63%) received adjuvant CAPOX. The remaining patients received capecitabine, 5-FU, or FOLFOX. Adjuvant chemotherapy was administered to 68% of SCRT patients and 82% of LCCRT patients (chi-squared test, P = 0.096). All patients in the LCCRT cohort received concurrent chemoradiation with capecitabine. The number of neoadjuvant chemotherapy and CAPOX cycles was similar between the SCRT and LCCRT groups (rank sum, P = 0.583 and P = 0.135, respectively). SCRT patients received significantly more cycles of adjuvant chemotherapy and adjuvant CAPOX (rank sum P = 0.007 and P = 0.028, respectively).

In 115 of 144 (80%) patients who underwent curative surgery, the R0 resection rate was high and comparable between groups (**Table 2**). Sphincter-preserving surgery was performed in 75% of SCRT patients and 76% of LCCRT patients (*P* = 0.865). The median time from radiotherapy to surgery was 14 weeks (IQR; 11–17 weeks) for SCRT and 18 weeks (IQR; 16–29 weeks) for LCCRT.

**Table 2:** Pathologic assessment and response among patients with a curative resection and disease-related treatment failures, 2013–2021.

Patients with a curative resection (N = 115)	SCRT (n = 60)	LCCRT (n = 55)	P^*^
**Tumor grade, n (%)**			0.197
Well differentiated	1 (2)	4 (7)	
Moderately differentiated	47 (78)	35 (64)	
Poorly differentiated	0	1 (2)	
No tumor or not assessable	12 (20)	15 (27)	
**Resection margin, n (%)**			0.567
R0	55 (92)	50 (91)	
R1	5 (8)	4 (7)	
R2	0	1 (2)	
**ypT, n (%)**			0.562
ypT0	9 (15)	8 (15)	
ypTis	0	1 (2)	
ypT1	1 (1)	2 (4)	
ypT2	16 (27)	8 (15)	
ypT3	31 (52)	33 (60)	
ypT4	3 (5)	3 (5)	
**ypN, n (%)**			0.959
ypN0	44 (73)	40 (73)	
ypN1	11 (18)	11 (20)	
ypN2	5 (8)	4 (7)	
**yp stage, n (%)**			0.819
0	7 (12)	7 (13)	
I	14 (23)	9 (16)	
II	23 (38)	24 (44)	
III	16 (27)	15 (27)	
**Pathologic complete response, n (%)**	7 (12)	10 (18)	0.325
**T-downstaging only, n (%)**	28 (47)	27 (49)	0.795
**N-downstaging only, n (%)**	39 (65)	36 (65)	0.959
**Both T- and N-downstaging, n (%)**	49 (82)	44 (80)	0.820
**Significant downstaging (ypT0-2N0), n (%)**	21 (35)	16 (29)	0.498

All eligible patients (N = 144)	SCRT (n = 78)	LCCRT (n = 66)	P^*^
**Disease-related treatment failure, first occurring, n (%)**	24 (31)	24 (36)	0.579
**Locoregional failure, n (%)** Local progression with unresectable tumor R2 resection pCR	9 (12) 4 (5) 0 0	12 (18) 7 (11) 1 (2) 0	
**Locoregional failure and distant metastasis, n (%)** Local progression with unresectable tumor R2 resection pCR	5 (6) 2 (3) 0 0	9 (14) 4 (6) 1 (2) 0	
**Distant metastasis, n (%)**	19 (24)	21 (32)	0.343
**New primary colorectal tumor, n (%)**	2 (3)	2 (3)	0.886
**Treatment-related death, n (%)**	0	1 (2)	0.275

LCCRT, long-course chemoradiotherapy; SCRT, short-course radiotherapy.

^*^Tested by Chi-squared test or Wilcoxon test for association.

### Treatment response

In the LCCRT group, 10 (18%) of 55 patients achieved pCR, compared to seven (12%) of 60 patients in the SCRT group (OR, 1.68; 95% CI, 0.59–4.78; P = 0.329; **Table 2**). Pathologic T-downstaging, N-downstaging, and significant pathologic downstaging (ypT0-2N0) were not significantly different between the two groups (**Supplementary Tables 1, 2**). Comparing the results of pre-treatment and post-treatment MRI, the percentage of patients considered radiological node-negative changed from 8% in the SCRT group and 9% in the LCCRT group before treatment to 34% and 45%, respectively (P <0.001). Post-neoadjuvant therapy MRI showed that 98% of SCRT and 93% of LCCRT patients achieved complete or partial response (**Table 3**, *P* = 0.175). For all 144 patients, the response rates were 77% (LCCRT) and 76% (SCRT) (*P* = 0.818).

**Table 3:** MRI radiological assessment and response comparing staging MRI and preoperative MRI, 2013–2021 (N = 115).

MRI radiological responses, n (%)^*^	SCRT (n = 60)	LCCRT (n = 55)	P^**^
Complete response	0 (0)	0 (0)	0.175
Partial response	59 (98)	51 (93)	
Stable disease	0 (0)	3 (5)	
Progressive disease	1 (2)	1 (2)	
Objective response^†^	59 (98)	51 (93)	

LCCRT, long-course chemoradiotherapy; MRI, magnetic resonance imaging; SCRT, short-course radiotherapy.

^*^Included patients who underwent surgical operation for rectal cancer.

^**^Tested by Fisher’s exact test for association.

^†^Objective response includes complete and partial responders.

### Survival outcomes

#### Disease-related treatment failure

**Figure 1:**
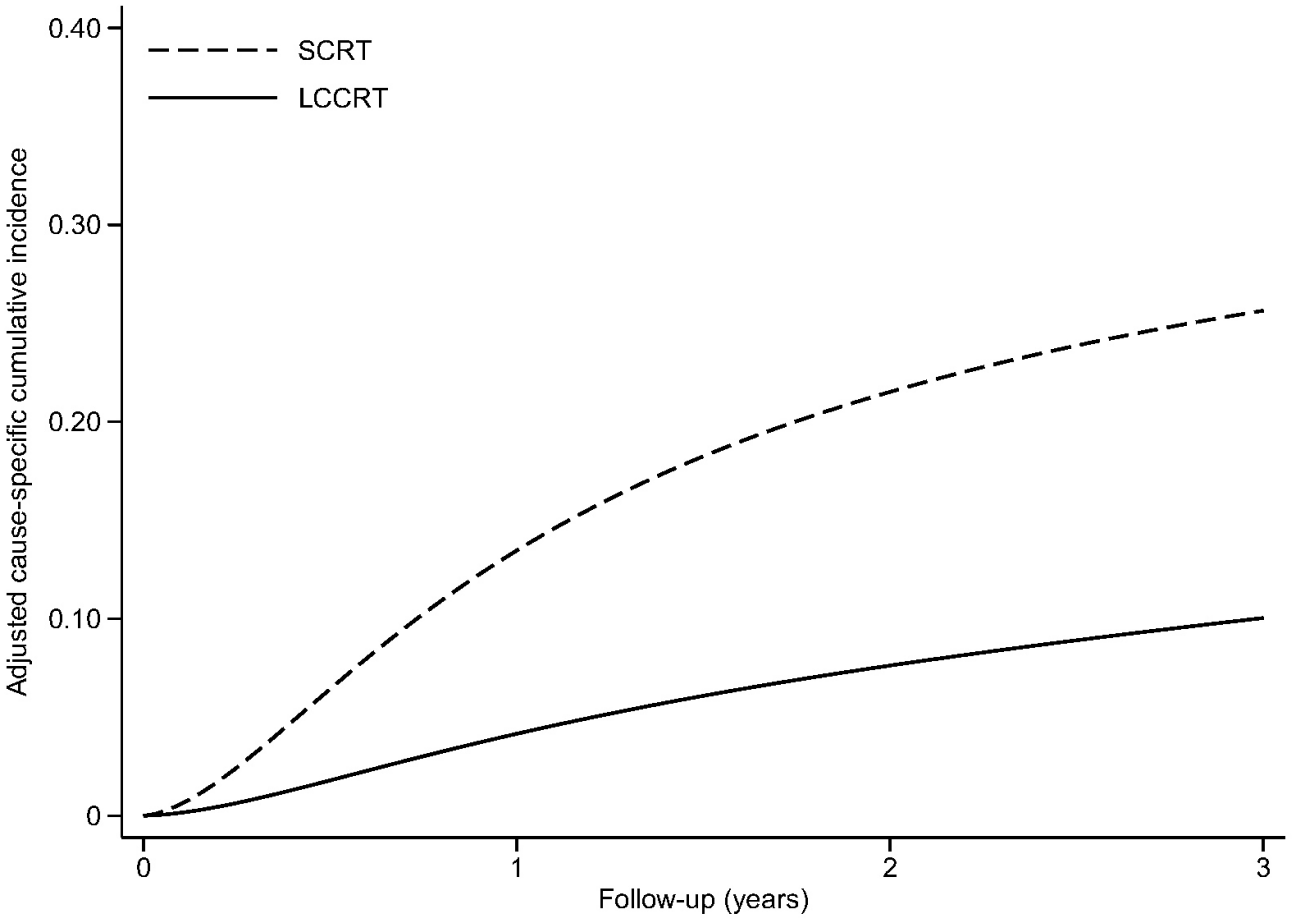
Adjusted cause-specific cumulative incidence of disease-related treatment failure. LCCRT, neoadjuvant chemoradiation; SCRT, short-course radiotherapy.

Thirty-two patients experienced disease-related treatment failure. The 1-, 2-, and 3-year cumulative probabilities of disease-related treatment failure were 4.2% (95% CI, 1.3%–12.0%), 7.6% (95% CI, 3.2%–18.3%), and 10.0% (95% CI, 4.5%–22.5%) for LCCRT and 13.5% (95% CI, 7.0%–26.1%), 21.5% (95% CI, 13.9%–33.4%), and 25.6% (95% CI, 17.0%–38.7%) for SCRT (**Figure 1**). Distant metastasis accounted for most failures (**Table 2**). LCCRT significantly lowered the risk of disease-related treatment failure (HR 0.26; 95% CI, 0.08%–0.87; P = 0.029; **Table 4** and **Figure 1**). Other factors included adjuvant chemotherapy (HR, 0.14; 95% CI, 0.03–0.69; P = 0.015), and age (HR per 1 increase, 0.93; 95% CI, 0.87–0.99; P = 0.032).

**Table 4:** Univariable and multivariable analyses of prognostic factors for disease-related treatment failure, 2013–2021 (N = 144).

Variables	Disease-related treatment failure			
	Univariable analysis		Multivariable analysis	
	HR (95% CI)	*P*	HR (95% CI)	*P*
**Treatment regimen (LCCRT vs. SCRT)**	0.99 (0.26–3.80)	0.990	0.26 (0.08–0.87)	0.029
**Age (per 1 year increase)**	0.97 (0.92–1.02)	0.262	0.93 (0.87–0.99)	0.032
**Sex (male vs. female)**	0.51 (0.18–1.47)	0.212	0.44 (0.07–2.82)	0.388
**ECOG performance status (2 vs. 0–1)**	3.05 (0.91–10.19)	0.071	2.08 (0.53–8.12)	0.292
Distance from the anal verge				
Mid rectum vs. low rectum High rectum vs. low rectum	0.48 (0.15–1.53) 2.51 (0.89–7.09)	0.217 0.082	0.56 (0.11–2.94) 0.37 (0.03–4.74)	0.494 0.445
**Extramural venous invasion on baseline MRI**	0.83 (0.18–3.83)	0.816	0.55 (0.09–3.35)	0.516
**Tumor grade (poorly/moderately vs. well differentiated)**	1.31 (0.43–4.02)	0.636	1.04 (0.33–3.23)	0.949
**Resection margin R1/R2 vs. R0**	4.03 (1.18–13.77)	0.026	1.55 (0.19–12.44)	0.683
**Cancer stage (III vs. II)**	1.35 (0.29–6.35)	0.706	1.84 (0.15–22.08)	0.631
**Significant downstaging (ypT0-2N0 vs. not)**	0.41 (0.09–1.91)	0.253	0.53 (0.08–3.62)	0.514
**Adjuvant chemotherapy (yes vs no)**	0.09 (0.03–0.29)	0.001	0.14 (0.03–0.69)	0.015

ECOG, Eastern Cooperative Oncology Group; HR, hazard ratio; MRI, magnetic resonance imaging; LCCRT, long-course chemoradiotherapy; SCRT, short-course radiotherapy.

#### Locoregional failure and distant metastasis

Twenty-one patients developed locoregional failure and 40 had distant metastases. The 1-, 2-, and 3-year cumulative probabilities of locoregional failure were 1.1% (95% CI, 0.2%–7.4%), 3.3% (95% CI, 0.7%–15.9%), and 4.2% (95% CI, 1.0%–18.3%) for LCCRT, and 3.2% (95% CI, 0.7%–15.3%), 8.8% (95% CI, 3.5%–22.3%), and 10.5% (95% CI, 4.4%–25.3%) for SCRT (HR 0.54; 95% CI, 0.12%–2.31%; P = 0.402) (**Supplementary Figure 1**). None of the factors were statistically significant (**Supplementary Table 3**).

The 1-, 2-, and 3-year cumulative probabilities of distant metastasis were 4.7% (95% CI, 1.8%–12.2%), 8.4% (95% CI, 3.9%–18.3%), and 10.7% (95% CI, 5.2%–21.9%) for LCCRT, and 11.6% (95% CI, 5.5%–24.5%), 18.6% (95% CI, 11.5%–30.0%), and 21.8% (95% CI, 13.8%–34.4%) for SCRT (HR 0.35; 95% CI, 0.11%–1.09%; P = 0.071) (**Supplementary Figure 2**). Adjuvant chemotherapy use (HR, 0.11; 95% CI, 0.03%–0.46%; P = 0.002) was associated with distant metastasis occurrence (**Supplementary Table 4**).

#### Overall survival

A total of 41 patients died during the study period. The 1-, 2-, and 3-year overall survival were 97.0% (95% CI, 88.4%–99.2%), 87.9% (95% CI, 77.2%–93.8%), and 78.7% (95% CI, 66.6%–86.8%) for LCCRT, and 91.0% (95% CI, 82.1%–95.6%), 82.6% (95% CI, 71.0%–89.9%), and 65.5% (95% CI, 47.7%–78.5%) for SCRT (HR, 0.35; 95% CI, 0.11%–1.17%; P = 0.090) (**Supplementary Figure 3**). Multivariable analysis showed that adjuvant chemotherapy (HR, 0.27; 95% CI, 0.09%–0.81%; P = 0.019) significantly affected overall survival (**Supplementary Table 5**).

### Toxicity

The toxicity events are shown in **Supplementary Tables 6, 7**. Grade ≥3 toxicities during neoadjuvant therapy occurred in 20% of the LCCRT patients and 23% of the SCRT patients. The most common preoperative grade ≥3 hematologic and non-hematologic toxicities were thrombocytopenia and gastrointestinal toxicities, respectively. Grade ≥3 postoperative complications occurred in 18% of the SCRT patients and 20% of the LCCRT patients. Two grade 5 postoperative complications in the LCCRT group involved anastomotic leakage and intra-abdominal infection, the latter being the most common grade ≥3 postoperative complications. No 30-day postoperative mortality occurred in either of the groups. Late rectal toxicity, the most common late toxicity, affected 15% of the LCCRT patients and 3% of the SCRT patients (P = 0.012) (**Supplementary Table 7**).

## Discussion

Combining LCCRT with multi-agent chemotherapy (induction or consolidative) before TME is feasible and enhances its effectiveness, as demonstrated by various phase 2 and randomized controlled trials (31–35). Several studies have compared SCRT plus neoadjuvant chemotherapy with standard chemoradiation followed by optional adjuvant chemotherapy in patients with locally advanced rectal cancer (6, 9, 10, 36, 37), showing improved or similar survival outcomes in the SCRT arm. However, whether adding preoperative systemic therapy to SCRT or LCCRT is better remains controversial, as fewer trials have investigated this approach. The ongoing ACO/ARO/AIO-18 trial (NCT04246684) compares SCRT and LCCRT followed by consolidative chemotherapy and surgery or selective organ preservation. Although retrospective, our study is the only one to examine the near-TNT approach with the sole variable being long-course versus SCR, while both arms received the same consolidative chemotherapy. This fills a knowledge gap and highlights the need for a prospective trial to address this question.

Our study compared survival outcomes and treatment responses in patients with locally advanced rectal cancer patients receiving consolidative chemotherapy with either LCCRT or SCRT before TME. The latter treatment approach was assessed in three large randomized controlled trials: Polish II, RAPIDO, and STELLAR (6, 9, 10, 37). We adjusted for potential confounding effects of various clinical, radiological, and pathological risk factors between the groups. Both treatment strategies, combined with consolidative chemotherapy before surgery, provided comparable downstaging effects and locoregional and distant control, while maintaining high tolerability and compliance. These findings offer further evidence to support modern neoadjuvant strategies in clinical practice.

An improved rate of disease-related treatment failure was observed in our LCCRT cohort, but differences in locoregional recurrence, distant metastasis, and overall survival were not demonstrated. This finding may be due to the increased power when analyzing disease-related treatment failure, a composite endpoint. PRODIGE-23, which mainly assesses the chemotherapy sequence, showed significant improvement in the 3-year distant metastasis rate for LCCRT after preoperative chemotherapy (33). Polish II and STELLAR demonstrated similar 3-year disease-free survival and locoregional recurrence rates between treatment arms (10, 37). Our LCCRT and SCRT arms achieved comparable survival outcomes, except for poorer overall survival, possibly related to patient selection heterogeneity, later lines of treatment received when disease progressed, and different neoadjuvant regimens (9, 33, 37). This provides external validity for our results. Considering our modest follow-up duration, a longer follow-up period (5–10 years) is needed to determine long-term survival and side effects. This is similar to the Polish II trial, in which overall survival benefit diminished after a median 8-year follow-up (6). Additionally, a recent report of the 5-year outcomes of RAPIDO showed higher locoregional recurrence in the SCRT arm, not revealed at 3 years, emphasizing the importance of long-term follow-up (38).

Adjuvant chemotherapy improves disease-related treatment failure, distant metastasis, and overall survival rates. Increased systemic treatment exposure may treat potential micrometastases and improves overall survival. Our study reported a distant metastasis rate of 27.7%, indicating the need for more effective systemic treatment. Evidence for adjuvant chemotherapy in rectal cancer comes largely from colon cancer experience, and its benefit after neoadjuvant treatment remains controversial (39). Further research should explore the role of adjuvant chemotherapy in the TNT era and optimize trimodality therapy use and sequence using risk-adapted strategies.

Our findings revealed a significant shift in the radiological nodal status from pre- to post-treatment. Notably, the LCCRT group exhibited a higher rate of achieving node-negative status post-treatment. Additionally, a considerable proportion of patients in both treatment groups, initially classified as node-positive following neoadjuvant therapy, subsequently attained ypN0 status. These observations underscore the intricacies involved in assessing treatment responses. Traditional static measurements of post-radiotherapy nodal status, classified simply as positive or negative, may not capture the full spectrum of temporal and volumetric tumor changes during treatment. Recognizing downstaging as a dynamic process influenced by both temporal progression and biological factors is vital. This dynamic nature of the tumor response calls for an augmented approach that includes both qualitative and quantitative evaluations. Advanced imaging modalities, such as serial MRI, which allows for the analysis of tumor volume alterations and activity changes in diffusion-weighted imaging, can be useful in providing a more holistic view of treatment effectiveness (40). This approach is particularly significant in the context of designing prospective studies. This highlights the necessity of comprehensive, multidimensional assessments to unravel the complex interactions between various treatment methods and tumor biology. Thus, our study emphasizes the importance of integrating both static and dynamic measurements in oncological research, enhancing our understanding of the varied responses of tumors to different treatment regimens.

### Toxicity and tolerance

Toxicity rates and postoperative complications were similar between the SCRT and LCCRT arms. Both treatment approaches were well tolerated, with comparable rates of grade ≥3 toxicities to other trials (24.2%–47.6%) (9, 10, 33, 37). However, the LCCRT group exhibited higher rates of late rectal toxicities. This contrasts with two studies that reported no significant difference in late toxicity between LCCRT and SCRT (3, 4). Long-term effects on functional outcomes and morbidities necessitate an extended follow-up.

### Strengths and limitations

Our study has several strengths, including the strict use of pelvic MRI as a standard staging tool to accurately define the extent of locoregional disease. In line with recent randomized controlled trials (9, 37), we utilized capecitabine as concurrent chemotherapy, which is non-inferior to 5-fluorouracil or capecitabine plus oxaliplatin and is more convenient for patients (41, 42). However, our study has some limitations. These include a modest follow-up period and inherent challenges associated with a single-institution retrospective cohort. Heterogeneity in chemotherapy regimens might have introduced confounding effects, although we endeavored to adjust for such factors. Additionally, we acknowledge the lack of explicit criteria for patient allocation to either the SCRT or LCCRT group as a limitation. The allocation process is influenced by a combination of clinical judgment, patient preferences, and logistical considerations at the time of treatment planning. This non-randomized approach might have introduced a selection bias, potentially affecting the comparability between groups and the generalizability of our findings. Although most patients had comparable baseline characteristics and were treated using contemporary radiotherapy techniques and consistent medical support within the same institution, the absence of a randomized allocation underscores the need for cautious interpretation of our results.

## Conclusion

Both LCCRT and SCRT, followed by consolidative chemotherapy and TME, are feasible strategies. Despite the higher late toxicity, this retrospective study suggests that the LCCRT approach may achieve better oncological outcomes than SCRT, warranting prospective confirmation. Future research should explore radiation dose–effect associations for tumor control and toxicity of radiotherapy and chemotherapy regimens, quality of MRI and histological assessment, quality of life, long-term survival outcomes, and local and distant relapse patterns.

## Data availability statement

The datasets presented in this article are not readily available because of patient privacy. Requests to access the datasets should be directed to the Hospital Authority, Hong Kong. Requests to access the datasets should be directed to https://www.ha.org.hk/.

## Ethics statement

The studies involving humans were approved by the Research Ethics Committee, New Territories West Cluster, Hospital Authority, Hong Kong (reference number: NTWC/REC/19054). The studies were conducted in accordance with local legislation and institutional requirements. The ethics committee/institutional review board waived the requirement for written informed consent for participation from the participants or the participants’ legal guardians/next of kin because this was a retrospective study with no expected harm to the subjects.

## Author contributions

SL: Conceptualization, Data curation, Formal analysis, Investigation, Methodology, Validation, Writing – original draft. PY: Conceptualization, Data curation, Formal analysis, Investigation, Methodology, Validation, Writing – original draft. BW: Investigation, Writing – original draft. NW: Investigation, Writing – original draft. BV: Investigation, Supervision, Writing – original draft. HM: Investigation, Supervision, Writing – original draft. FAL: Supervision, Writing – original draft.

## References

[1] Sauer R Becker H Hohenberger W Rödel C Wittekind C Fietkau R. Preoperative versus postoperative chemoradiotherapy for rectal cancer. New Engl J Med (2004) 351(17):1731–40. doi: 10.1056/NEJMoa040694 15496622

[2] Sebag-Montefiore D Stephens RJ Steele R Monson J Grieve R Khanna S. Preoperative radiotherapy versus selective postoperative chemoradiotherapy in patients with rectal cancer (MRC CR07 and NCIC-CTG C016): a multicentre, randomised trial. Lancet (2009) 373(9666):811–20. doi: 10.1016/S0140-6736(09)60484-0 PMC2668947 19269519

[3] Bujko K Nowacki MP Nasierowska-Guttmejer A Michalski W Bebenek M Kryj M. Long-term results of a randomized trial comparing preoperative short-course radiotherapy with preoperative conventionally fractionated chemoradiation for rectal cancer. Br J Surg (2006) 93(10):1215–23. doi: 10.1002/bjs.5506 16983741

[4] Ngan SY Burmeister B Fisher RJ Solomon M Goldstein D Joseph D. Randomized trial of short-course radiotherapy versus long-course chemoradiation comparing rates of local recurrence in patients with T3 rectal cancer: Trans-Tasman Radiation Oncology Group trial 01.04. J Clin Oncol (2012) 30(31):3827–33. doi: 10.1200/JCO.2012.42.9597 23008301

[5] Erlandsson J Holm T Pettersson D Berglund Å Cedermark B Radu C. Optimal fractionation of preoperative radiotherapy and timing to surgery for rectal cancer (Stockholm III): a multicentre, randomised, non-blinded, phase 3, non-inferiority trial. Lancet Oncol (2017) 18(3):336–46. doi: 10.1016/S1470-2045(17)30086-4 28190762

[6] Ciseł B Pietrzak L Michalski W Wyrwicz L Rutkowski A Kosakowska E. Long-course preoperative chemoradiation versus 5 × 5 Gy and consolidation chemotherapy for clinical T4 and fixed clinical T3 rectal cancer: long-term results of the randomized Polish II study. Ann Oncol (2019) 30(8):1298–303. doi: 10.1093/annonc/mdz186 31192355

[7] Myerson RJ Tan B Hunt S Olsen J Birnbaum E Fleshman J. Five fractions of radiation therapy followed by 4 cycles of FOLFOX chemotherapy as preoperative treatment for rectal cancer. Int J Radiat Oncol Biol Phys (2014) 88(4):829–36. doi: 10.1016/j.ijrobp.2013.12.028 PMC4028157 24606849

[8] Jin J Tang Y Hu C Cai Y Zhu Y Cheng G. A multicenter, randomized, phase III trial of short-term radiotherapy plus chemotherapy versus long-term chemoradiotherapy in locally advanced rectal cancer (STELLAR): The final reports. J Clin Oncol (2021) 39(15_suppl):3510–. doi: 10.1200/JCO.2021.39.15_suppl.3510 PMC9113208 35263150

[9] Bahadoer RR Dijkstra EA van Etten B Marijnen CAM Putter H Kranenbarg EM-K. Short-course radiotherapy followed by chemotherapy before total mesorectal excision (TME) versus preoperative chemoradiotherapy, TME, and optional adjuvant chemotherapy in locally advanced rectal cancer (RAPIDO): a randomised, open-label, phase 3 trial. Lancet Oncol (2021) 22(1):29–42. doi: 10.1016/S1470-2045(20)30555-6 33301740

[10] Bujko K Wyrwicz L Rutkowski A Malinowska M Pietrzak L Kryński J. Long-course oxaliplatin-based preoperative chemoradiation versus 5 × 5 Gy and consolidation chemotherapy for cT4 or fixed cT3 rectal cancer: results of a randomized phase III study. Ann Oncol (2016) 27(5):834–42. doi: 10.1093/annonc/mdw062 26884592

[11] Myerson RJ Garofalo MC El Naqa I Abrams RA Apte A Bosch WR. Elective clinical target volumes for conformal therapy in anorectal cancer: a radiation therapy oncology group consensus panel contouring atlas. Int J Radiat Oncol Biol Phys (2009) 74(3):824–30. doi: 10.1016/j.ijrobp.2008.08.070 PMC2709288 19117696

[12] Fernandez-Martos C Garcia-Albeniz X Pericay C Maurel J Aparicio J Montagut C. Chemoradiation, surgery and adjuvant chemotherapy versus induction chemotherapy followed by chemoradiation and surgery: long-term results of the Spanish GCR-3 phase II randomized trial†. Ann Oncol (2015) 26(8):1722–8. doi: 10.1093/annonc/mdv223 25957330

[13] Moore J Price T Carruthers S Selva-Nayagam S Luck A Thomas M. Prospective randomized trial of neoadjuvant chemotherapy during the ‘wait period’ following preoperative chemoradiotherapy for rectal cancer: results of the WAIT trial. Colorectal Dis (2017) 19(11):973–9. doi: 10.1111/codi.13724 28503826

[14] Kim SY Joo J Kim TW Hong YS Kim JE Hwang IG. A randomized phase 2 trial of consolidation chemotherapy after preoperative chemoradiation therapy versus chemoradiation therapy alone for locally advanced rectal cancer: KCSG CO 14-03. Int J Radiat Oncol Biol Phys (2018) 101(4):889–99. doi: 10.1016/j.ijrobp.2018.04.013 29976501

[15] Maréchal R Vos B Polus M Delaunoit T Peeters M Demetter P. Short course chemotherapy followed by concomitant chemoradiotherapy and surgery in locally advanced rectal cancer: a randomized multicentric phase II study. Ann Oncol (2012) 23(6):1525–30. doi: 10.1093/annonc/mdr473 22039087

[16] Edge SB Compton CC. The American Joint Committee on Cancer: the 7th edition of the AJCC cancer staging manual and the future of TNM. Ann Surg Oncol (2010) 17(6):1471–4. doi: 10.1245/s10434-010-0985-4 20180029

[17] Amin MB Greene FL Edge SB Compton CC Gershenwald JE Brookland RK. The Eighth Edition AJCC Cancer Staging Manual: Continuing to build a bridge from a population-based to a more “personalized” approach to cancer staging. CA: A Cancer J Clin (2017) 67(2):93–9. doi: 10.3322/caac.21388 28094848

[18] Dindo D Demartines N Clavien PA. Classification of surgical complications: a new proposal with evaluation in a cohort of 6336 patients and results of a survey. Ann Surg (2004) 240(2):205–13. doi: 10.1097/01.sla.0000133083.54934.ae PMC1360123 15273542

[19] Fokas E Glynne-Jones R Appelt A Beets-Tan R Beets G Haustermans K. Outcome measures in multimodal rectal cancer trials. Lancet Oncol (2020) 21(5):e252–e64. doi: 10.1016/S1470-2045(20)30024-3 32359501

[20] Andersen PK Geskus RB de Witte T Putter H. Competing risks in epidemiology: possibilities and pitfalls. Int J Epidemiol. (2012) 41(3):861–70. doi: 10.1093/ije/dyr213 PMC3396320 22253319

[21] Kipourou DK Charvat H Rachet B Belot A. Estimation of the adjusted cause-specific cumulative probability using flexible regression models for the cause-specific hazards. Stat Med (2019) 38(20):3896–910. doi: 10.1002/sim.8209 PMC6771712 31209905

[22] Gray RJ. A class of K-sample tests for comparing the cumulative incidence of a competing risk. Ann Statistics. (1988) 16(3):1141–54. doi: 10.1214/aos/1176350951

[23] Geskus RB. Data analysis with competing risks and intermediate states. Boca Raton: CRC Press (2016).

[24] Armitage P Berry G Matthews JNS. Statistical methods in medical research. Wiley: Wiley-Blackwell (2008).

[25] Shen W Ning J Yuan Y. A direct method to evaluate the time-dependent predictive accuracy for biomarkers. Biometrics (2015) 71(2):439–49. doi: 10.1111/biom.12293 PMC4479968 25758584

[26] Chesnaye NC Stel VS Tripepi G Dekker FW Fu EL Zoccali C. An introduction to inverse probability of treatment weighting in observational research. Clin Kidney J (2021) 15(1):14–20. doi: 10.1093/ckj/sfab158 35035932 PMC8757413

[27] Kaplan EL Meier P. Nonparametric estimation from incomplete observations. J Am Stat Assoc (1958) 53(282):457–81. doi: 10.1080/01621459.1958.10501452

[28] Mantel N. Evaluation of survival data and two new rank order statistics arising in its consideration. Cancer Chemother Rep (1966) 50(3):163–70. 5910392

[29] StataCorp. Stata statistical software: release 16. College Station, TX: StataCorp LP (2019).

[30] Royston P. Flexible parametric alternatives to the Cox model: update. Stata J (2004) 4(1):98–101. doi: 10.1177/1536867X0100400112

[31] Chua YJ Barbachano Y Cunningham D. Neoadjuvant capecitabine and oxaliplatin before chemoradiotherapy and total mesorectal excision in MRI-defined poor-risk rectal cancer: a phase 2 trial. Lancet Oncol (2010) 11(3):241–8. doi: 10.1016/S1470-2045(09)70381-X 20106720

[32] Schou JV Larsen FO Rasch L Linnemann D Langhoff J Høgdall E. Induction chemotherapy with capecitabine and oxaliplatin followed by chemoradiotherapy before total mesorectal excision in patients with locally advanced rectal cancer. Ann Oncol (2012) 23(10):2627–33. doi: 10.1093/annonc/mds056 22473488

[33] Conroy T Bosset J-F Etienne P-L Rio E François É Mesgouez-Nebout N. Neoadjuvant chemotherapy with FOLFIRINOX and preoperative chemoradiotherapy for patients with locally advanced rectal cancer (UNICANCER-PRODIGE 23): a multicentre, randomised, open-label, phase 3 trial. Lancet Oncol (2021) 22(5):702–15. doi: 10.1016/S1470-2045(21)00079-6 33862000

[34] Petrelli F Trevisan F Cabiddu M Sgroi G Bruschieri L Rausa E. Total neoadjuvant therapy in rectal cancer: A systematic review and meta-analysis of treatment outcomes. Ann surgery. (2020) 271(3):440–8. doi: 10.1097/SLA.0000000000003471 31318794

[35] Riesco-Martinez MC Fernandez-Martos C Gravalos-Castro C Espinosa-Olarte P La Salvia A Robles-Diaz L. Impact of total neoadjuvant therapy vs. Standard chemoradiotherapy in locally advanced rectal cancer: A systematic review and meta-analysis of randomized trials. Cancers (Basel) (2020) 12(12):3655. doi: 10.3390/cancers12123655 33291454 PMC7762140

[36] Markovina S Youssef F Roy A Aggarwal S Khwaja S DeWees T. Improved metastasis- and disease-free survival with preoperative sequential short-course radiation therapy and FOLFOX chemotherapy for rectal cancer compared with neoadjuvant long-course chemoradiotherapy: results of a matched pair analysis. Int J Radiat Oncol Biol Phys (2017) 99(2):417–26. doi: 10.1016/j.ijrobp.2017.05.048 28871992

[37] Jin J Tang Y Hu C Jiang L-M Jiang J Li N. Multicenter, randomized, phase III trial of short-term radiotherapy plus chemotherapy versus long-term chemoradiotherapy in locally advanced rectal cancer (STELLAR). J Clin Oncol (2022) 40(15):1681–92. doi: 10.1200/JCO.21.01667 PMC9113208 35263150

[38] Dijkstra EA Nilsson PJ Hospers GAP Bahadoer RR Meershoek-Klein Kranenbarg E Roodvoets AGH. Locoregional failure during and after short-course radiotherapy followed by chemotherapy and surgery compared to long-course chemoradiotherapy and surgery - A five-year follow-up of the RAPIDO trial. Ann surgery. (2023) 278(4):e766–72. doi: 10.1097/SLA.0000000000005799 PMC10481913 36661037

[39] Breugom AJ Swets M Bosset J-F Collette L Sainato A Cionini L. Adjuvant chemotherapy after preoperative (chemo)radiotherapy and surgery for patients with rectal cancer: a systematic review and meta-analysis of individual patient data. Lancet Oncol (2015) 16(2):200–7. doi: 10.1016/S1470-2045(14)71199-4 25589192

[40] Chandramohan A Siddiqi UM Mittal R Eapen A Jesudason MR Ram TS. Diffusion weighted imaging improves diagnostic ability of MRI for determining complete response to neoadjuvant therapy in locally advanced rectal cancer. Eur J Radiol Open (2020) 7:100223. doi: 10.1016/j.ejro.2020.100223 32140502 PMC7044654

[41] Hofheinz RD Wenz F Post S Matzdorff A Laechelt S Hartmann JT. Chemoradiotherapy with capecitabine versus fluorouracil for locally advanced rectal cancer: a randomised, multicentre, non-inferiority, phase 3 trial. Lancet Oncol (2012) 13(6):579–88. doi: 10.1016/S1470-2045(12)70116-X 22503032

[42] Schmoll HJ Stein A Van Cutsem E Price T Hofheinz RD Nordlinger B. Pre- and postoperative capecitabine without or with oxaliplatin in locally advanced rectal cancer: PETACC 6 trial by EORTC GITCG and ROG, AIO, AGITG, BGDO, and FFCD. J Clin Oncol (2021) 39(1):17–29. doi: 10.1200/JCO.20.01740 33001764

