# Supplementary material for: Neoadjuvant short-course radiotherapy or chemoradiation plus consolidative chemotherapy followed by radical operation for locally advanced rectal cancer
Source: Front Oncol. 2024 Jan 23;13:1284569. doi: 10.3389/fonc.2023.1284569 (PMC10844885; doi:10.3389/fonc.2023.1284569)
Supplement: Supplementary file 1 [file DataSheet_1.pdf]

Figure S1. Adjusted cause-specific cumulative incidence of locoregional failure, Hong Kong, 2013–2021 (N = 144). Abbreviations: LCCRT, long-course chemoradiotherapy; SCRT, short-course radiotherapy.

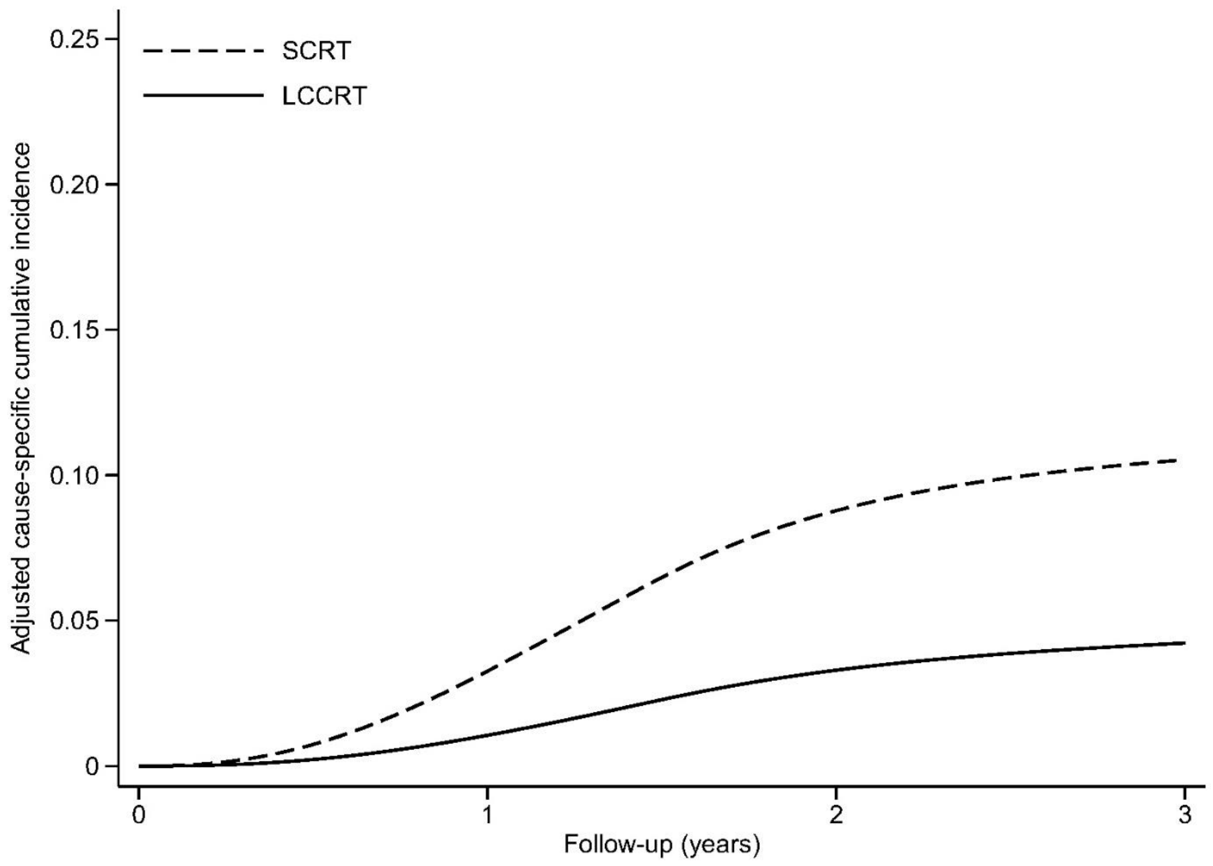

Figure S2. Adjusted cause-specific cumulative incidence of distant metastasis, Hong Kong, 2013–2021 (N = 144). Abbreviations: LCCRT, long-course chemoradiotherapy; SCRT, short-course radiotherapy.

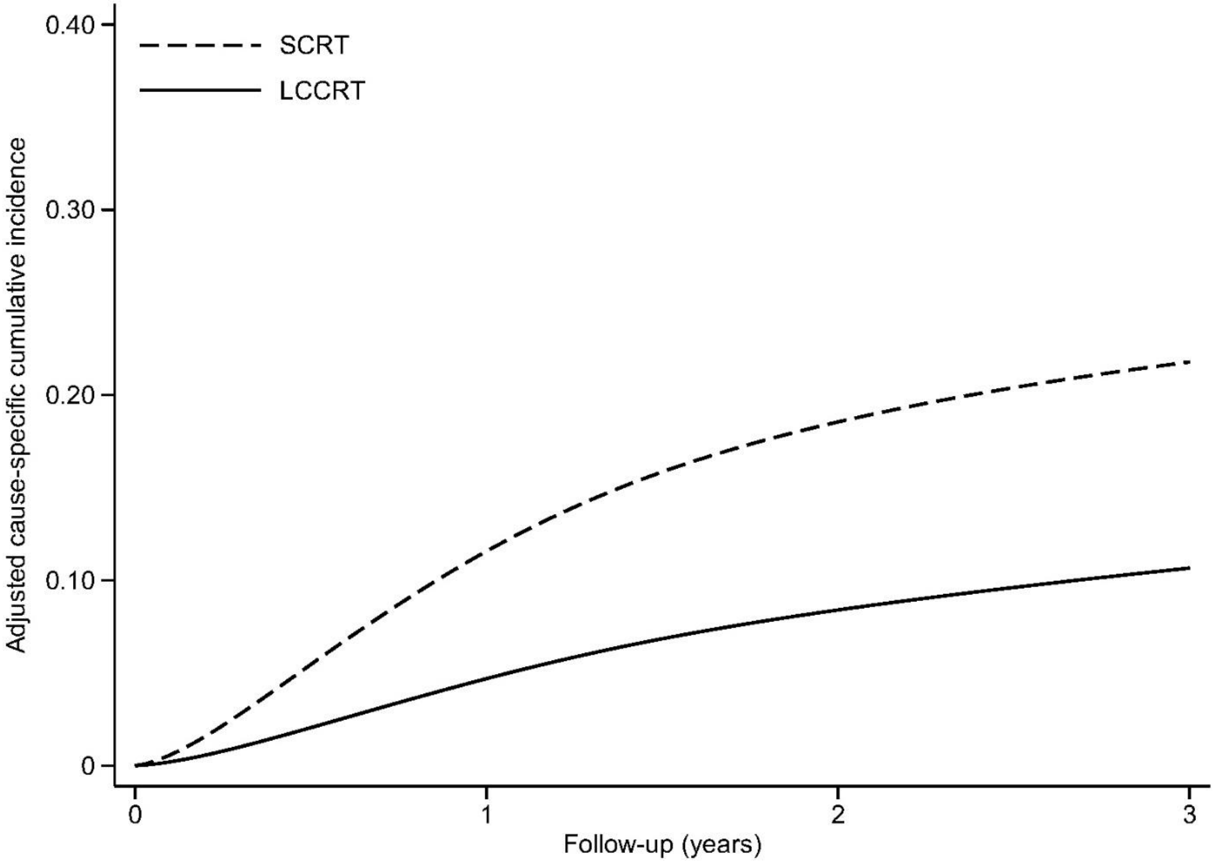

Figure S3. Kaplan-Meier overall survival curve, Hong Kong, 2013–2021 (N = 144). Abbreviations: LCCRT, long-course chemoradiotherapy; SCRT, short-course radiotherapy.

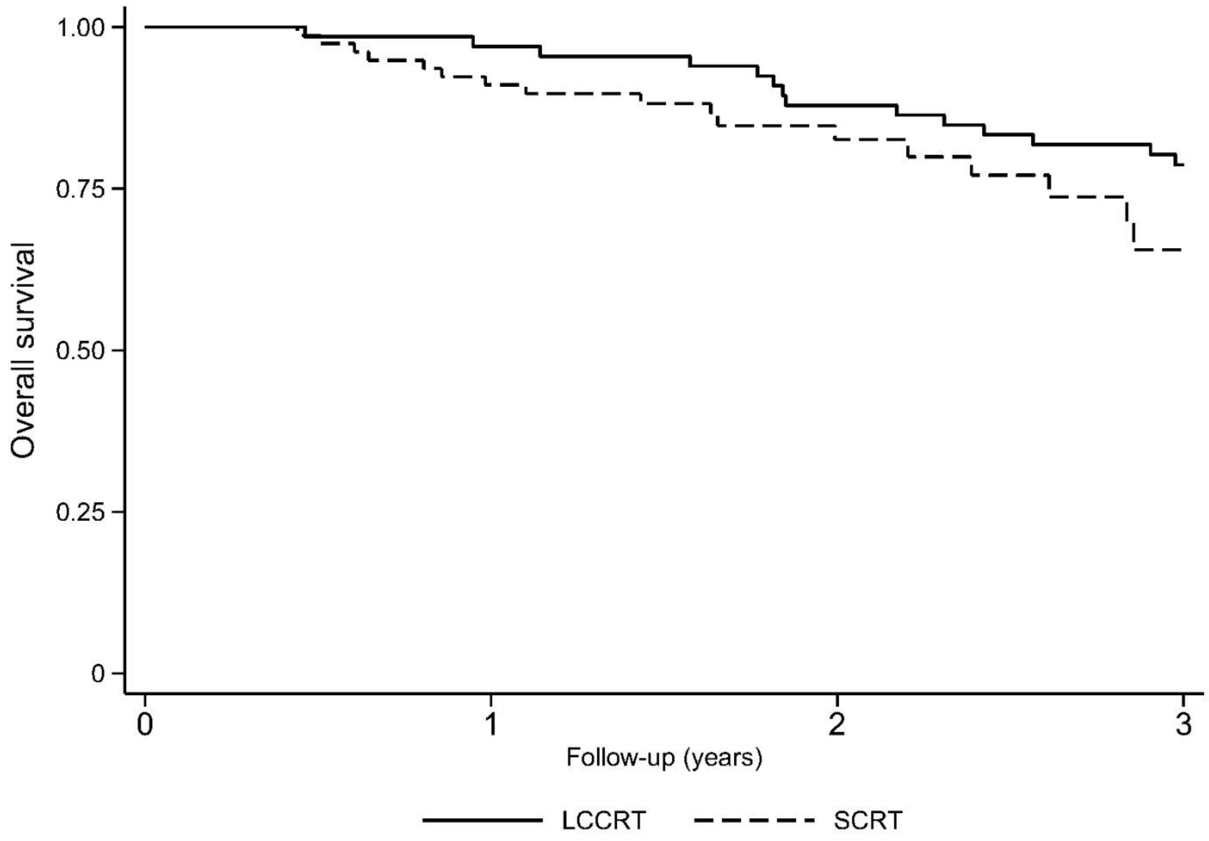

Table S1. Distribution of MRI radiological and pathologic response in the SCRT group (n = 60).

Baseline MRI clinical staging	Pathological T staging					Pathological N staging		
	ypT0	ypT1	ypT2	ypT3	ypT4	ypN0	ypN1	ypN2
cT3	8 (13%)	1 (2%)	15 (25%)	29 (48%)	1 (2%)	–	–	–
cT4	1 (2%)	0	1 (2%)	2 (3%)	2 (3%)	–	–	–
Node negative	–	–	–	–	–	5 (8%)	0	0
Node positive	–	–	–	–	–	39 (65%)	11 (18%)	5 (8%)
**Restaging MRI clinical staging**	**Pathological T staging**					**Pathological N staging**		
	ypT0	ypT1	ypT2	ypT3	ypT4	ypN0	ypN1	ypN2
cT0	0	0	1 (2%)	0	0	–	–	–
cT1	0	0	0	0	0	–	–	–
cT2	2 (3%)	0	6 (10%)	2 (3%)	0	–	–	–
cT3	6 (10%)	1 (2%)	9 (15%)	28 (47%)	2 (3%)	–	–	–
cT4	1 (2%)	0	0	1 (2%)	1 (2%)	–	–	–
Node negative	–	–	–	–	–	18 (30%)	1 (2%)	1 (2%)
Node positive	–	–	–	–	–	26 (43%)	10 (17%)	4 (7%)

Abbreviations: MRI, magnetic resonance imaging; SCRT, short-course radiotherapy.

Table S2. Distribution of MRI radiological and pathologic response in the LCCRT group (n = 55).

Baseline MRI clinical staging	Pathological T staging					Pathological N staging		
	ypT0	ypT1	ypT2	ypT3	ypT4	ypN0	ypN1	ypN2
cT3	7* (13%)	1 (2%)	5 (9%)	25 (45%)	0	–	–	–
cT4	2 (4%)	1 (2%)	3 (5%)	8 (15%)	3 (5%)	–	–	–
Node negative	–	–	–	–	–	4 (7%)	1 (2%)	0
Node positive	–	–	–	–	–	36 (65%)	10 (18%)	4 (7%)
**Restaging MRI clinical staging**	**Pathological T staging**					**Pathological N staging**		
	ypT0	ypT1	ypT2	ypT3	ypT4	ypN0	ypN1	ypN2
cT0	0	0	0	0	0	–	–	–
cT1	0	0	0	0	0	–	–	–
cT2	0	0	1 (2%)	1 (2%)	0	–	–	–
cT3	7* (13%)	2 (4%)	5 (9%)	24 (44%)	0	–	–	–
cT4	2 (4%)	0	2 (4%)	8 (15%)	3 (5%)	–	–	–
Node negative	–	–	–	–	–	21 (38%)	3 (5%)	1 (2%)
Node positive	–	–	–	–	–	19 (35%)	8 (15%)	3 (5%)

Abbreviations: MRI, magnetic resonance imaging; LCCRT, long-course chemoradiotherapy.

*Included one patient with ypTis disease.

Table S3. Univariable and multivariable analyses of prognostic factors for locoregional failure, Hong Kong, 2013–2021 (N = 144).

	**Locoregional failure**			
	**Univariable analysis**		**Multivariable analysis***	
**Variables**	HR (95% CI)	*P*	HR (95% CI)	*P*
**Treatment regimen (LCCRT vs. SCRT)**	1.15 (0.48–2.78)	0.754	0.54 (0.12–2.31)	0.402
**Sex (male vs. female)**	1.93 (0.45–8.28)	0.377	1.60 (0.17–14.74)	0.680
**ECOG performance status (2 vs. 0–1)**	1.39 (0.47–4.12)	0.557	0.42 (0.03–6.22)	0.526
**Distance from the anal verge**				
Mid rectum vs. low rectum	1.11 (0.40–3.07)	0.833	0.79 (0.17–3.77)	0.772
High rectum vs. low rectum	2.74 (0.84–8.98)	0.096	1.22 (0.16–9.18)	0.849
**Extramural venous invasion on baseline MRI**	2.31 (0.95–5.59)	0.064	1.89 (0.43–8.28)	0.398
**Resection margin R1/R2 vs. R0**	4.66 (1.20–18.05)	0.026	3.29 (0.69–15.63)	0.134
**Cancer stage (III vs. II)**	0.78 (0.23–2.63)	0.684	0.41 (0.03–4.90)	0.478
**Significant downstaging (ypT0-2N0 vs. not)**	0.12 (0.02–0.93)	0.042	0.30 (0.03–2.59)	0.273
**Adjuvant chemotherapy (yes vs no)**	0.15 (0.05–0.40)	<0.001	0.40 (0.09–1.69)	0.212

Abbreviations: ECOG, Eastern Cooperative Oncology Group; HR, hazard ratio; MRI, magnetic resonance imaging; LCCRT, long-course chemoradiotherapy; SCRT, short-course radiotherapy.

*Age and tumor grade were dropped due to non-convergence of the model.

Table S4. Univariable and multivariable analyses of prognostic factors for distant metastasis, Hong Kong, 2013–2021 (N = 144).

	**Distant metastasis**			
	**Univariable analysis**		**Multivariable analysis***	
**Variables**	HR (95% CI)	*P*	HR (95% CI)	*P*
**Treatment regimen (LCCRT vs. SCRT)**	0.96 (0.25–3.67)	0.952	0.35 (0.11–1.09)	0.071
**Age (per 1 year increase)**	0.97 (0.91–1.02)	0.233	0.93 (0.87–1.00)	0.044
**Sex (male vs. female)**	0.39 (0.14–1.13)	0.083	0.44 (0.06–3.17)	0.413
**ECOG performance status (2 vs. 0–1)**	2.27 (0.67–7.72)	0.189	1.34 (0.38–4.71)	0.649
**Distance from the anal verge**				
Mid rectum vs. low rectum	0.58 (0.18–1.88)	0.366	0.46 (0.07–2.90)	0.412
High rectum vs. low rectum	3.18 (1.08–9.32)	0.035	0.32 (0.02–5.50)	0.433
**Extramural venous invasion on baseline MRI**	1.08 (0.33–3.60)	0.897	0.25 (0.01–4.42)	0.345
**Tumor grade (poorly/moderately vs. well differentiated)**	1.62 (0.15–17.08)	0.689	0.72 (0.21–2.40)	0.589
**Resection margin R1/R2 vs. R0**	3.19 (0.87–11.73)	0.081	1.51 (0.12–19.52)	0.754
**Significant downstaging (ypT0-2N0 vs. not)**	0.52 (0.11–2.47)	0.410	0.59 (0.09–4.02)	0.588
**Adjuvant chemotherapy (yes vs no)**	0.12 (0.04–0.40)	<0.001	0.11 (0.03–0.46)	0.002

Abbreviations: ECOG, Eastern Cooperative Oncology Group; HR, hazard ratio; MRI, magnetic resonance imaging; LCCRT, long-course chemoradiotherapy; SCRT, short-course radiotherapy.

*Cancer stage was omitted because most of the patients with distant metastases had stage III disease.

Table S5. Univariable and multivariable analyses of prognostic factors for overall survival, Hong Kong, 2013–2021 (N = 144).

	Overall survival			
	**Univariable analysis**		**Multivariable analysis**	
**Variables**	HR (95% CI)	*P*	HR (95% CI)	*P*
**Treatment regimen (LCCRT vs. SCRT)**	0.59 (0.30–1.14)	0.118	0.35 (0.11–1.17)	0.090
**Age (per 1 year increase)**	1.00 (0.97–1.03)	0.816	0.97 (0.91–1.02)	0.249
**Sex (male vs. female)**	1.28 (0.50–3.30)	0.603	1.91 (0.23–15.75)	0.847
**ECOG performance status (2 vs. 0–1)**	2.95 (1.48–5.88)	0.002	1.44 (0.32–6.46)	0.632
**Distance from the anal verge**				
Mid rectum vs. low rectum	1.12 (0.52–2.43)	0.771	1.20 (0.31–4.71)	0.789
High rectum vs. low rectum	2.79 (1.13–6.87)	0.026	1.46 (0.20–10.51)	0.710
**Extramural venous invasion on baseline MRI**	1.16 (0.53–2.54)	0.711	0.97 (0.26–3.59)	0.960
**Tumor grade (poorly/moderately vs. well differentiated)**	1.67 (0.22–12.45)	0.619	1.24 (0.13–11.61)	0.848
**Resection margin R1/R2 vs. R0**	2.35 (0.87–6.31)	0.091	1.78 (0.41–7.79)	0.443
**Cancer stage (III vs. II)**	1.42 (0.44–4.64)	0.557	1.61 (0.16–15.85)	0.681
**Significant downstaging (ypT0-2N0 vs. not)**	0.32 (0.11–0.89)	0.029	1.68 (0.35–8.12)	0.519
**Adjuvant chemotherapy (yes vs no)**	0.16 (0.07–0.33)	<0.001	0.27 (0.09–0.81)	0.019

Abbreviations: ECOG, Eastern Cooperative Oncology Group; HR, hazard ratio; LCCRT, long-course chemoradiotherapy; MRI, magnetic resonance imaging; SCRT, short-course radiotherapy.

Table S6. Preoperative and postoperative toxicities for all patients, Hong Kong, 2013–2021 (N = 144).

**Toxicities**	**Toxicity grades**						
	**SCRT (n = 78)**			**LCCRT (n = 66)**			
	**3**	**4**	**5**	**3**	**4**	**5**	***P***
**Preoperative toxicities, n (%)**							
Gastrointestinal	4 (5)	0	0	2 (3)	0	0	
Genitourinary	1 (1)	0	0	1 (2)	0	0	
Neutropenia	1 (1)	0	0	2 (3)	0	0	
Anemia	6 (8)	0	0	4 (6)	0	0	
Thrombocytopenia	4 (5)	0	0	1 (2)	0	0	
Other non-hematologic	2 (3)	0	0	3 (5)	0	0	
Total	18 (23)	0	0	13 (20)	0	0	0.687
**Postoperative complications, n (%)**							
Anastomotic leakage	0	1 (1)	0	2 (3)	0	1 (2)	
Intraabdominal infection	2 (3)	3 (4)	0	2 (3)	0	1 (2)	
Wound healing problem	2 (3)	1 (1)	0	2 (3)	0	0	
Ileus	1 (1)	1 (1)	0	1 (2)	0	0	
Others	1 (1)	0	0	3 (5)	1 (2)	0	
Total	6 (8)	6 (8)	0	10 (15)	1 (2)	2 (3)	0.066

Abbreviations: LCCRT, long-course chemoradiotherapy; SCRT, short-course radiotherapy

Table S7. Late toxicities for all patients, Hong Kong, 2013–2021 (N = 144).

**Rectal toxicity, n (%)**	**SCRT (n = 78)**	**LCCRT (n = 66)**	***P***
Hemorrhage	0	1 (2)	
Pain	1 (1)	1 (2)	
Ulcer	0	0	
Fecal incontinence	0	3 (5)	
Fistulation or perforation	0	2 (3)	
Stenosis	1 (1)	1 (2)	
Others	0	2 (3)	
Total	2 (3)	10 (15)	0.012
**Small bowel toxicity, n (%)**			
Intestinal obstruction	1 (1)	0	
Diarrhea	0	1 (2)	
Ulcer	0	0	
Fistulation or perforation	0	0	
Others	0	0	
Total	1 (1)	1 (2)	1.000
**Bladder toxicity, n (%)**			
Hematuria	1 (1)	1 (2)	
Pain	0	0	
Ulcer	0	0	
Incontinence	0	0	
Fistulation or perforation	0	1 (2)	
obstruction/ stenosis	0	0	
Others	0	0	
Total	1 (1)	2 (3)	0.5930

Abbreviations: LCCRT, long-course chemoradiotherapy; SCRT, short-course radiotherapy
